# Author Correction: The impact of extreme heat on lake warming in China

**DOI:** 10.1038/s41467-024-44936-6

**Published:** 2024-01-17

**Authors:** Weijia Wang, Kun Shi, Xiwen Wang, Yunlin Zhang, Boqiang Qin, Yibo Zhang, R. Iestyn Woolway

**Affiliations:** 1 Taihu Laboratory for Lake Ecosystem Research, State Key Laboratory of Lake Science and Environment, Nanjing Institute of Geography and Limnology, Chinese Academy of Sciences, Nanjing, 210008 China; 2 University of Chinese Academy of Sciences, 100049 Beijing, China; 3 College of Nanjing, University of Chinese Academy of Sciences, Nanjing, 211135 China; 4 School of Geography & Ocean Science, Nanjing University, Nanjing, China; 5 School of Ocean Sciences, Bangor University, Menai Bridge, Anglesey, UK

**Keywords:** Limnology, Limnology

Correction to: *Nature Communications* 10.1038/s41467-023-44404-7, published online 02 January 2024

The original version of this Article contained two errors in Fig. 2. In Fig. 2g the color legends for the “Original” and “After removal of heat extremes” probability densities were reversed. The correct legend shows “Original” in red and “After removal of heat extremes” in cyan color. In Fig. 2h the horizontal axis of the plot was incorrectly titled “Trend of SAT (°C/decade)”, while the correct title is “Trend of LSWT (°C/decade)”. These have been corrected in both the PDF and HTML versions of the Article.

