# Supplementary material for: The impact of extreme heat on lake warming in China
Source: Nat Commun. 2024 Jan 2;15:70. doi: 10.1038/s41467-023-44404-7 (PMC10762129; doi:10.1038/s41467-023-44404-7)
Supplement: Supplementary file 1 — Supplementary Information [file 41467_2023_44404_MOESM1_ESM.pdf]

Supplementary Information

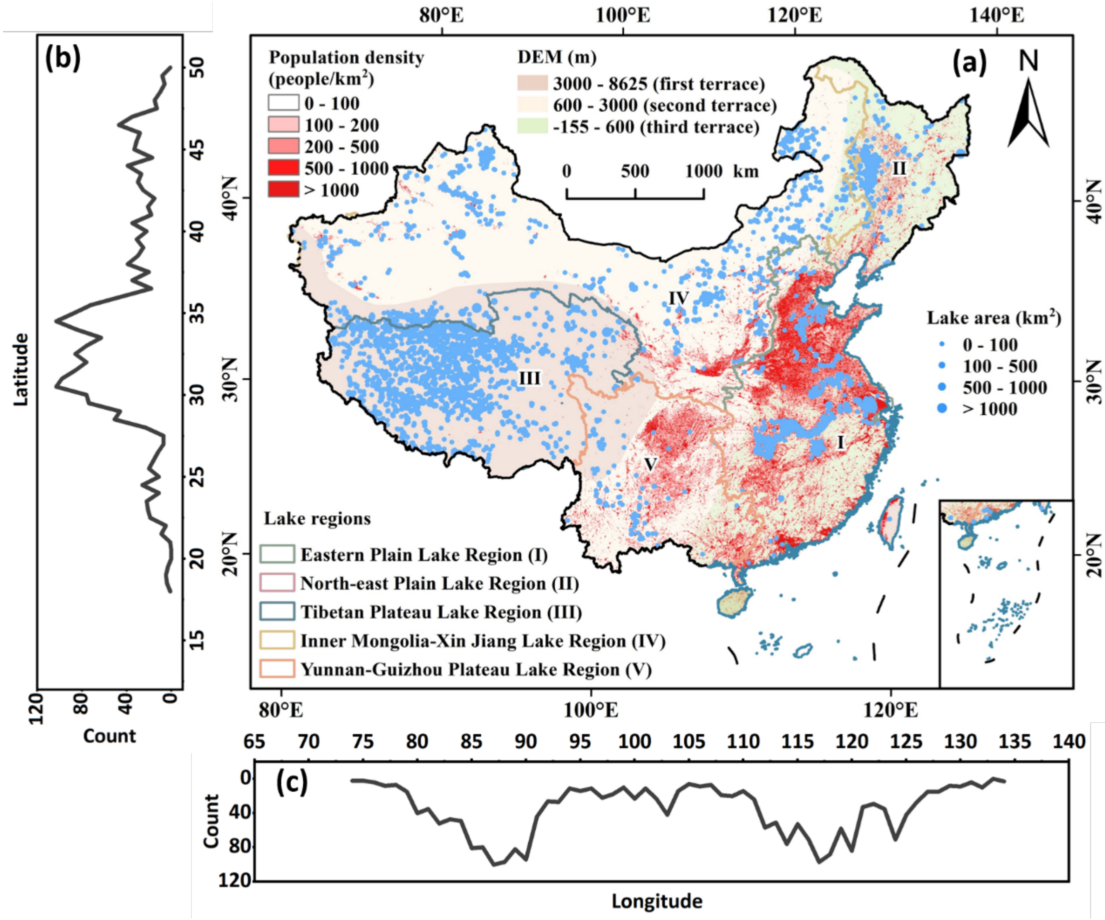

Supplementary Figure 1| Geographic characteristics of the study area. a, Map of studied Lakes. The area of lakes is denoted by the size of circles. The five lake regions in China, namely Eastern Plain Lake Region (I), North-east Plain Lake Region (II), Tibetan Plateau Lake Region (III), Inner Mongolia-Xin Jiang Lake Region (IV) and Ynnan-Guizhou Plateau Lake Region (V), are indicated by different colored borders. The three terraces in China according to elevation are indicated by brown, yellow and green color blocks, respectively. Population density is distinguished by different red blocks. **b** and **c** are the counts of lakes along latitude and longitude, respectively.

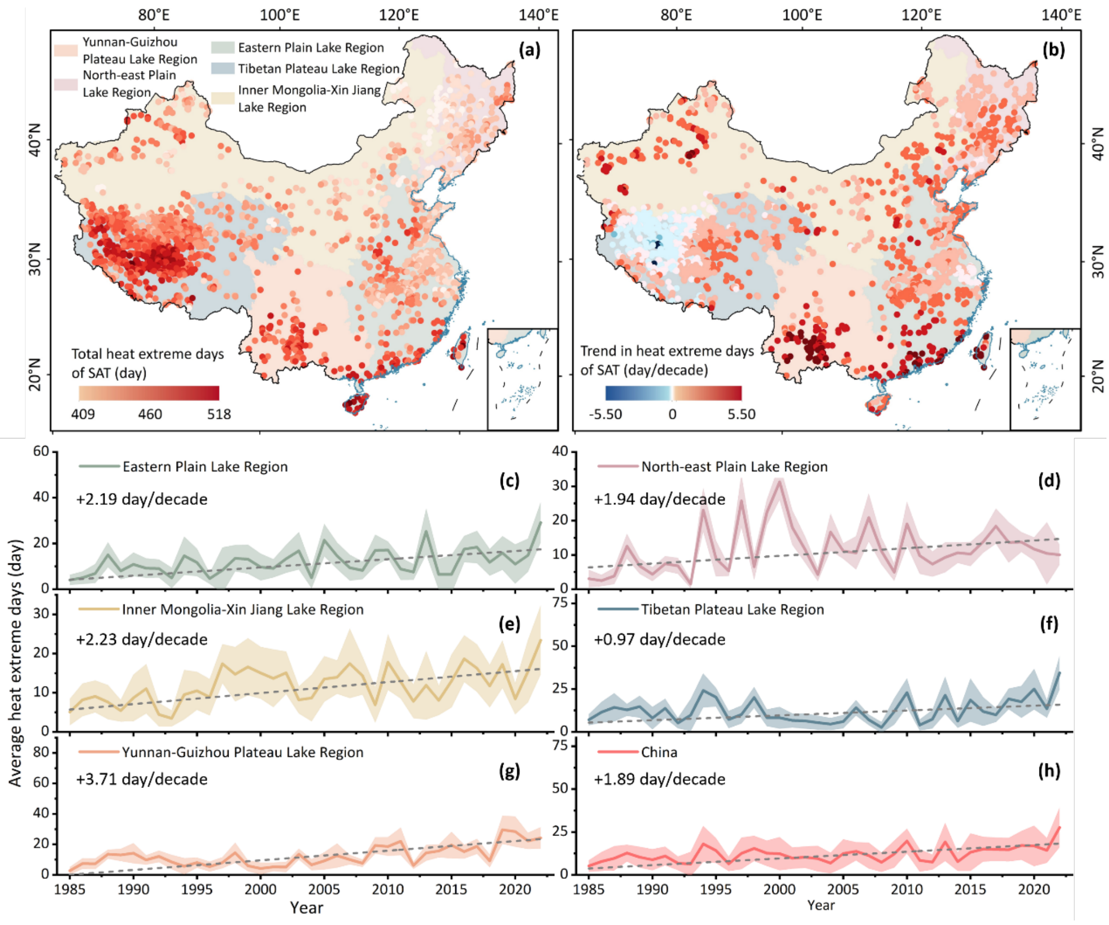

Supplementary Figure 2| Total heat extreme days and trend in heat extreme days of lake surface air temperature (SAT). a, Total heat extreme days of SAT between 1985 and 2022. **b**, Trend in heat extreme days of SAT. Annual summer average heat extreme days of SAT in Eastern Plain Lake Region **(c)**, North-east Plain Lake Region **(d)**, Inner Mongolia-Xin Jiang Lake Region **(e)**, Tibetan Plateau Lake Region **(f)**, Yunnan-Guizhou Plateau Lake Region **(g)** and China **(h)** are represented by curves, shaded with standard deviation. Source data are provided as a Source Data file.

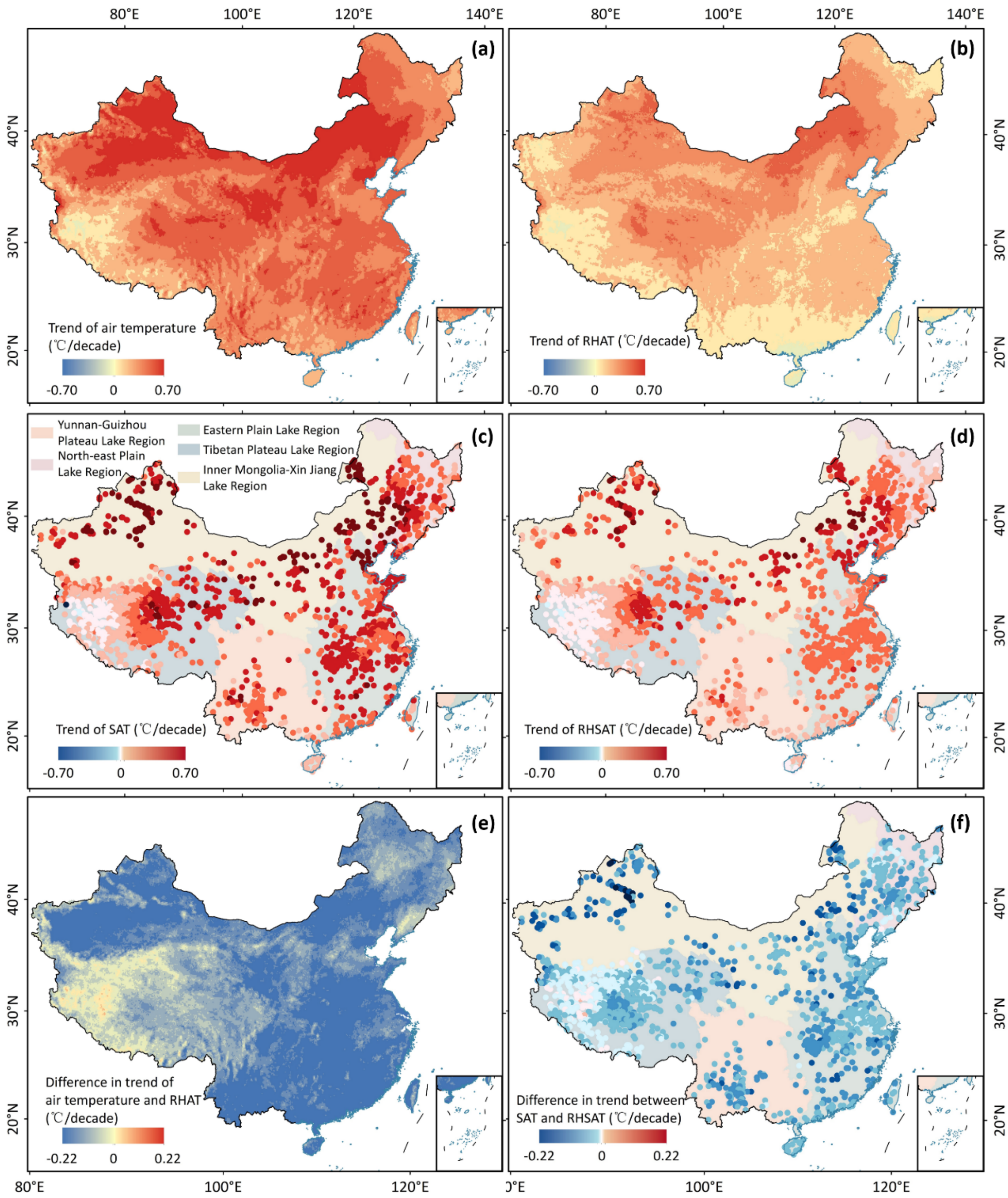

Supplementary Figure 3| Trend of air temperature and lake surface air temperature (SAT). a, Trend of air temperature. **b**, Trend of air temperature after removal of heat extremes (RHAT). **c**, Trend of SAT. **d**, Trend of SAT after removal of heat extremes (RHSAT). **e**, Difference in trend of air temperature and RHAT. **f**, Difference in trend of SAT and RHSAT. Source data are provided as a Source Data file.

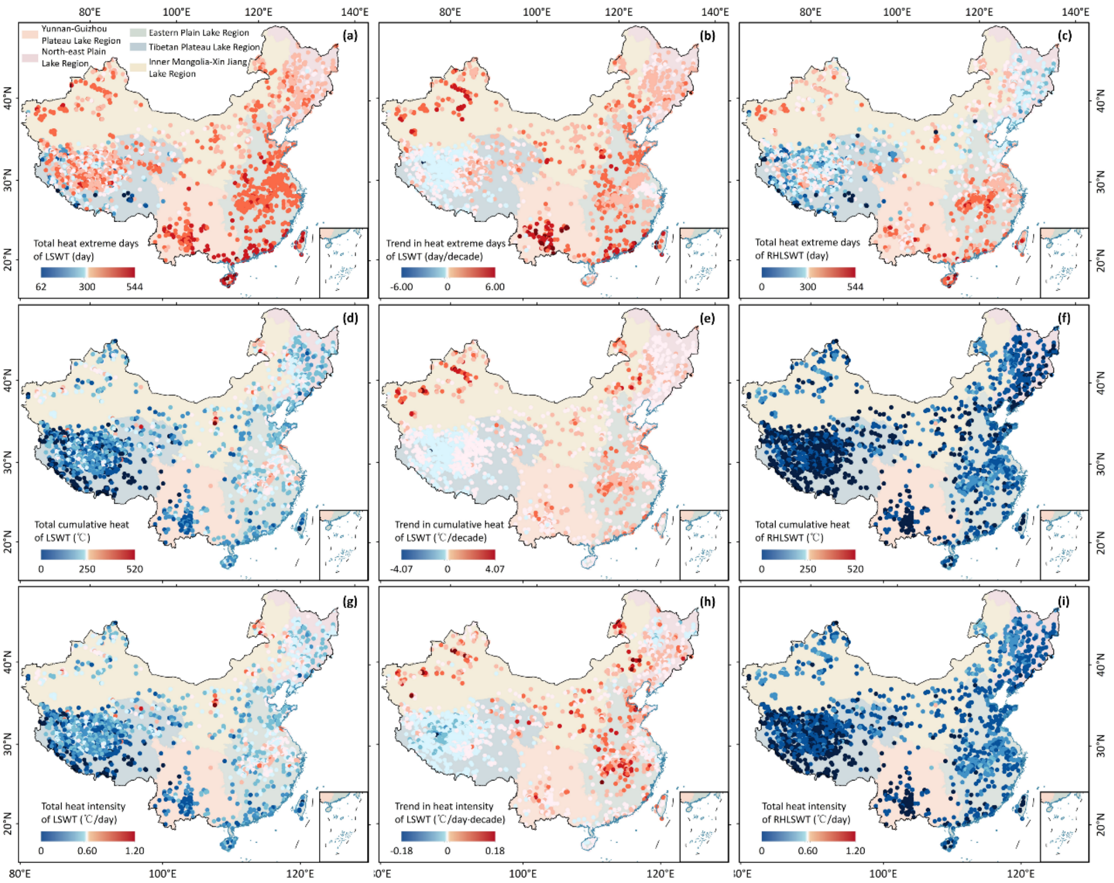

Supplementary Figure 4| Comparison of heat extremes of lake surface water temperature (LSWT) before and after removal of heat extremes in lake surface air temperature. a, d and **g** denote the total number of days, total cumulative heat, and average intensity of heat extremes of LSWT from 1985 to 2022, respectively; **b, e** and **h** represent the trends in days of heat extremes, annual cumulative heat, and annual average intensity, respectively; **c, f** and **i** indicate the total number of days, total cumulative heat, and average intensity of heat extremes of LSWT calculated from the threshold of original LSWT after removing the heat extremes of air temperature, respectively. Source data are provided as a Source Data file.

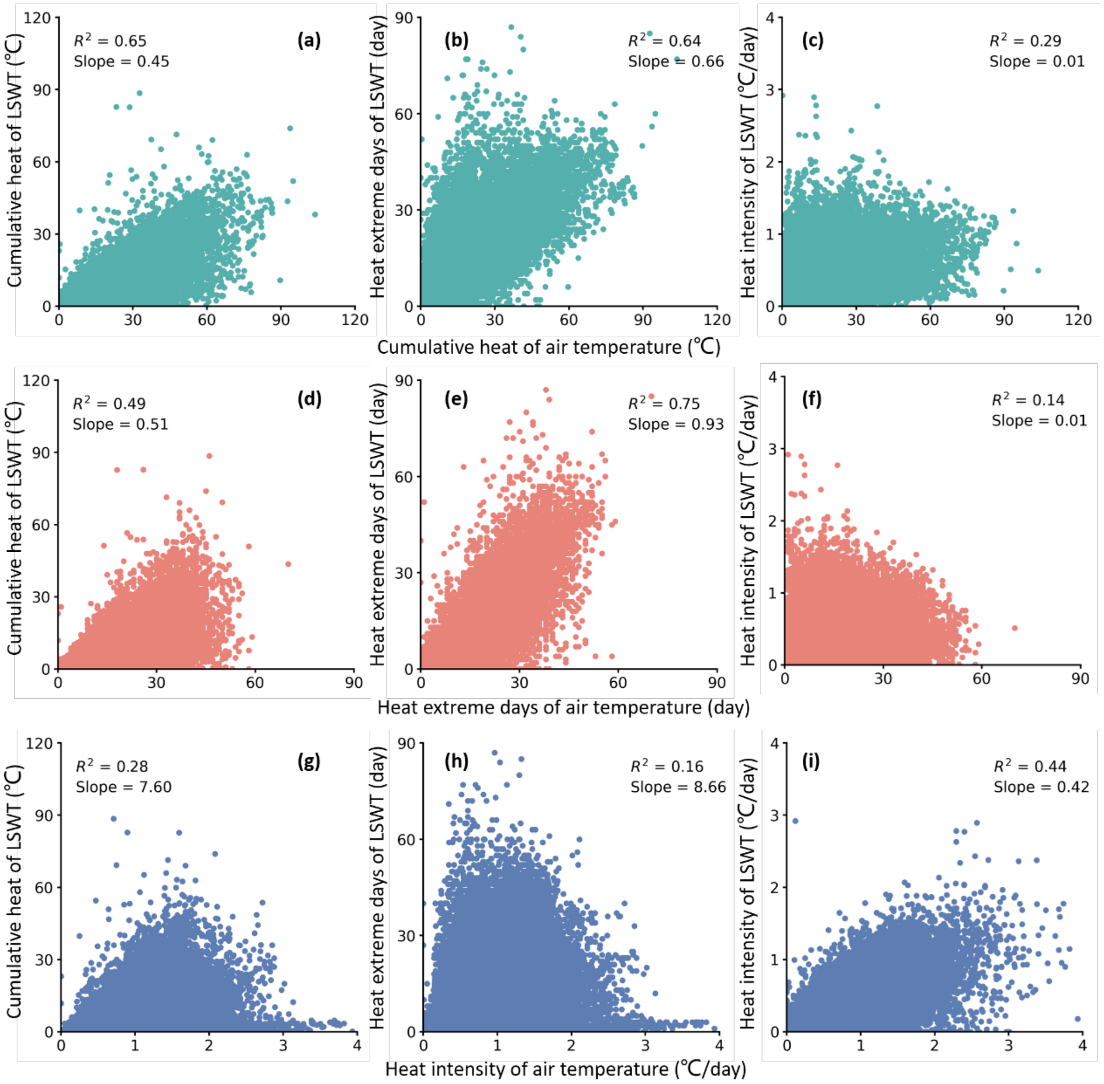

Supplementary Figure 5| Sensitivity of heat extremes of LSWT to heat extremes of air temperature. a-c, Comparison of the three elements of heat extremes (cumulative heat, days of heat extremes and heat intensity) of LSWT with the cumulative heat change of air temperature. **d-f**, Comparison of the three elements of heat extremes of LSWT with days of heat extremes of air temperature. **g-i**, Comparison of the three elements of heat extremes of LSWT with days of heat intensity of air temperature. Source data are provided as a Source Data file.

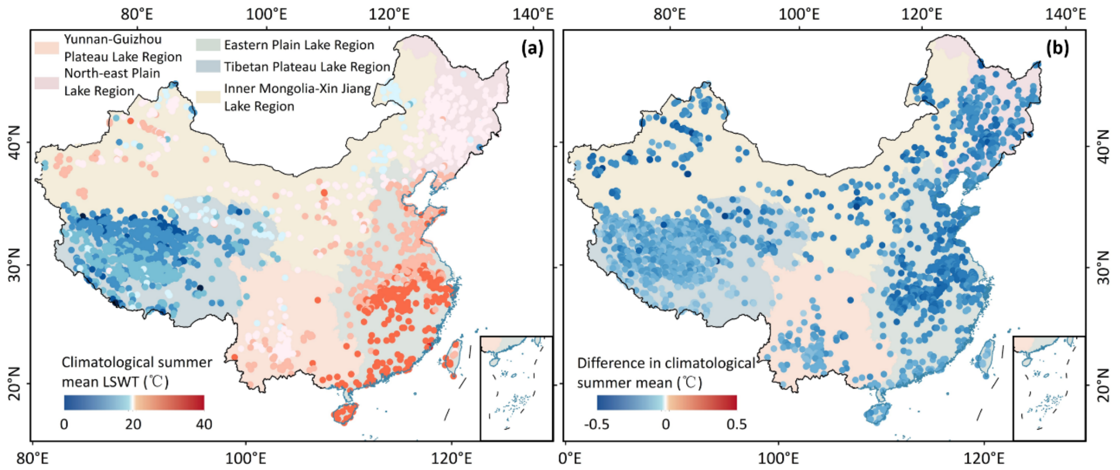

Supplementary Figure 6| Climatological summer mean of LSWT and LSWT simulated by removing heat extremes from SAT in Air2Water (RHLSWT). a, Climatological summer mean of LSWT. **b**, Difference in climatological summer mean between LSWT and RHLSWT. Source data are provided as a Source Data file.

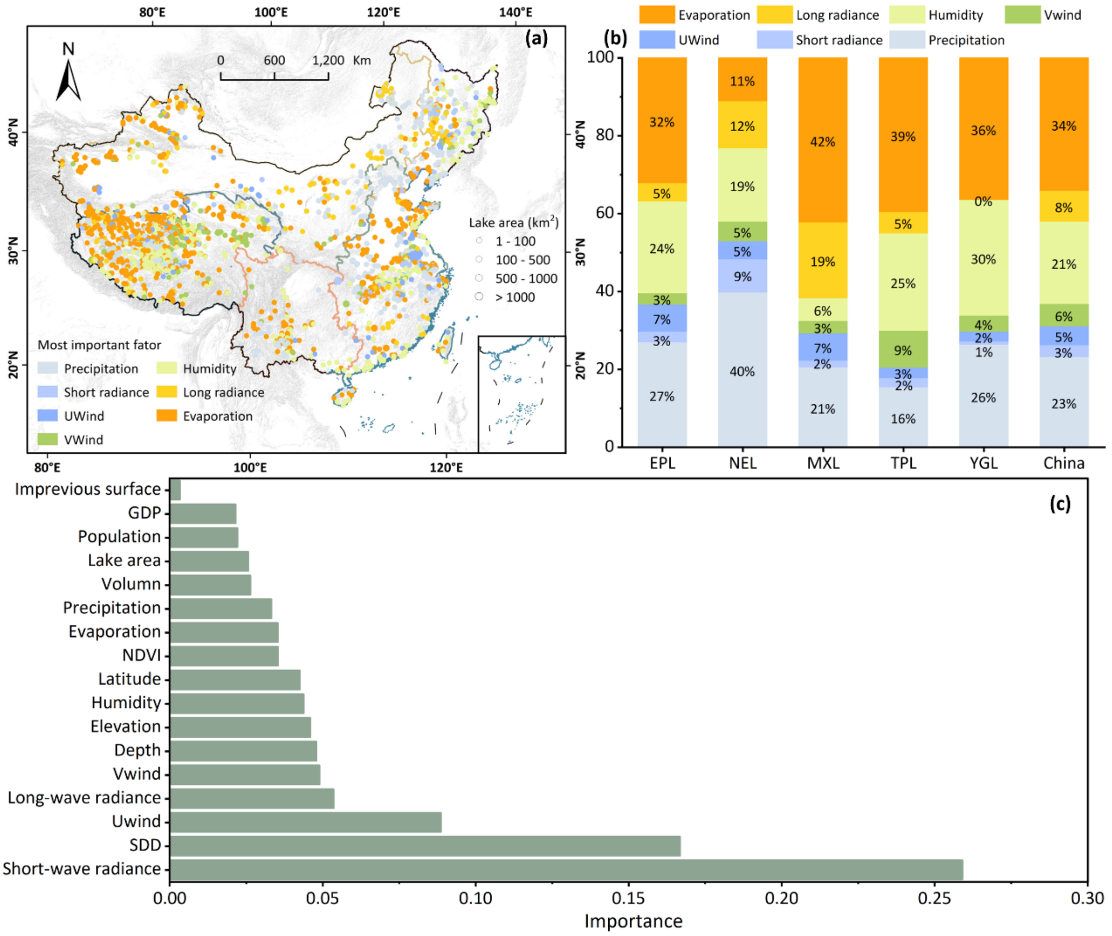

Supplementary Figure 7| Driving Factors. a, The most important meteorological factor influencing the variation of the difference between interannual LSWT and LSWT after removal of heat extremes (RHLSWT) for each lake. **b**, Proportions of lakes with the most important meteorological factor at the five lake regions and national scales. **c**, Importance of factors influencing the contribution of heat extremes to LSWT. Source data are provided as a Source Data file.

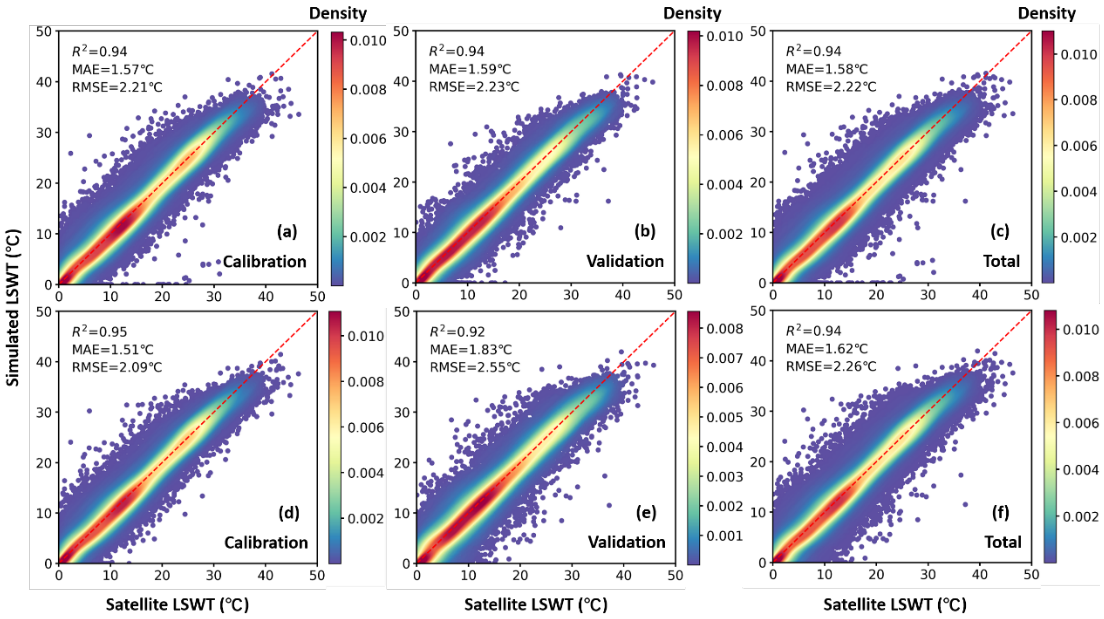

Supplementary Figure 8| Comparison of LSWT between simulated data from Air2Water and Satellite observations. a-c are the LSWT simulated by investing all the data from Landsat 8 between 2013 and 2019 into the Air2Water model, **d-f** are the LSWT simulated by using data from Landsat 8 between 2013 and 2017, and the remaining satellite data of 2018-2019 were used to verify the simulation accuracy. **a** and **d** are comparisons of satellite data in 2013-2017 and the simulated LSWT; **b** and **e** represent comparisons of satellite data in 2018-2019 and their simulated results, respectively; **c** and **f** are comparisons of all satellite data and their simulated LSWT. Source data are provided as a Source Data file.

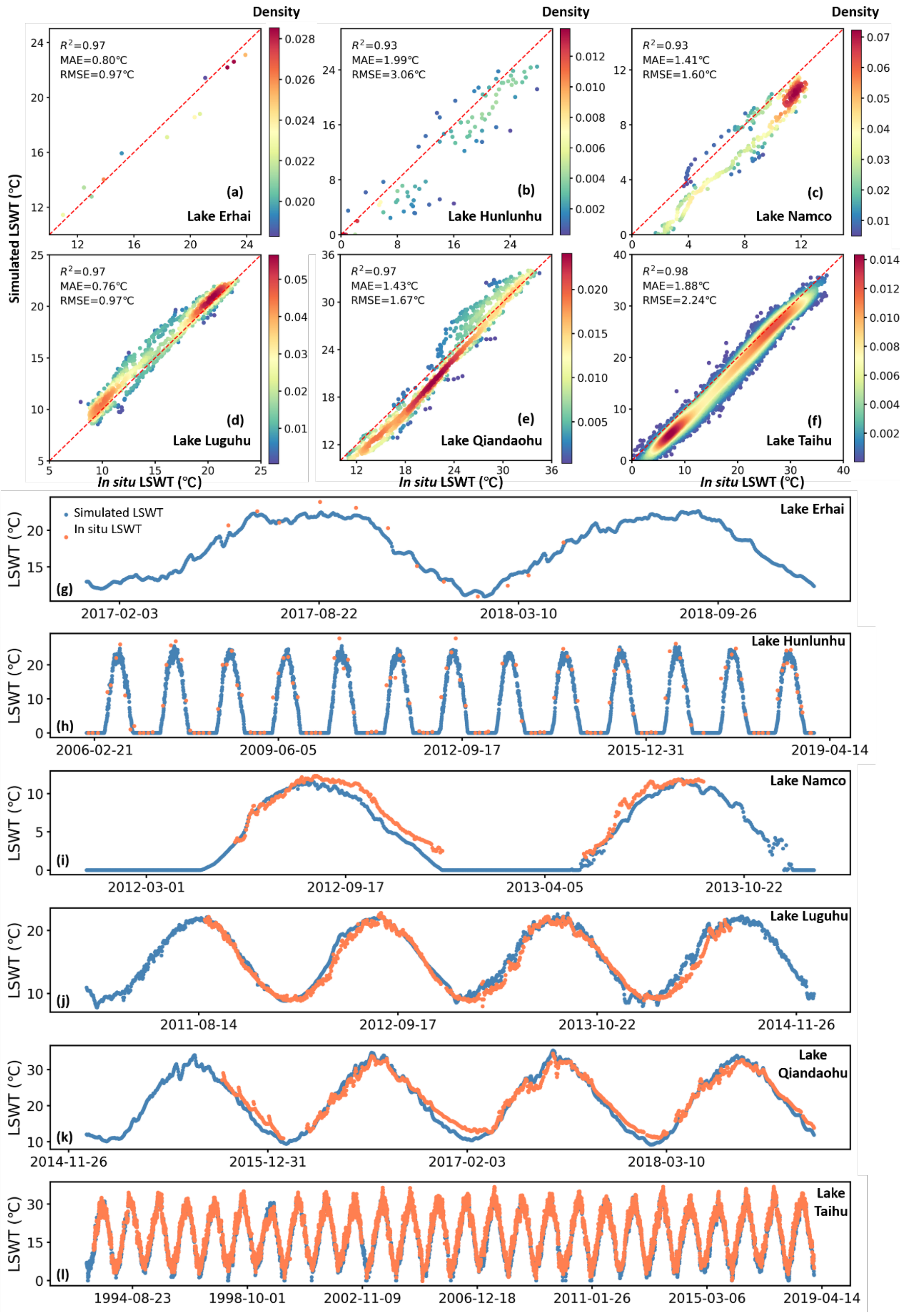

Supplementary Figure 9| Comparison of LSWT between simulated data from Air2Water and *in situ* observations. a, Lake Erhai. **b**, Lake Hulunhu. **c**, Lake Namco. **d**, Lake Luguhu. **e**

Lake Qiandaohu. **f**, Lake Taihu. **g-l,** Comparison in time series of LSWT, corresponding to the lakes of a1-a6. Source data are provided as a Source Data file.

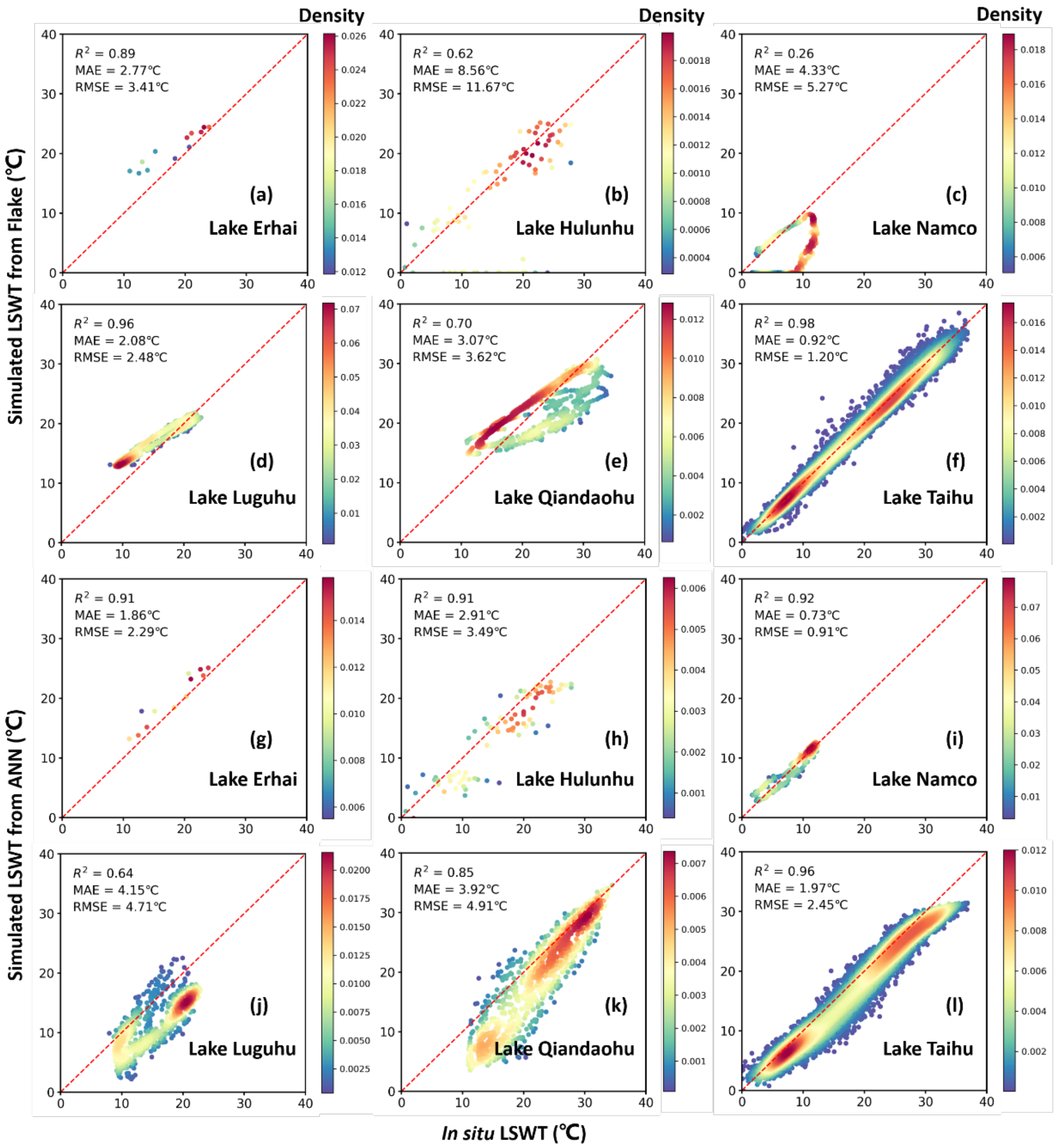

Supplementary Figure 10| Comparison of the accuracy of Flake and ANN. A-f, Comparison of LSWT between simulated data from Flake and *in situ* observations in Lake Erhai, Lake Hulunhu, Lake Namco, Lake Luguhu, Lake Qiandaohu and Lake Taihu. **g-l** are comparisons of LSWT between simulated data from ANN and *in situ* observations in above six lakes. Source data are provided as a Source Data file.

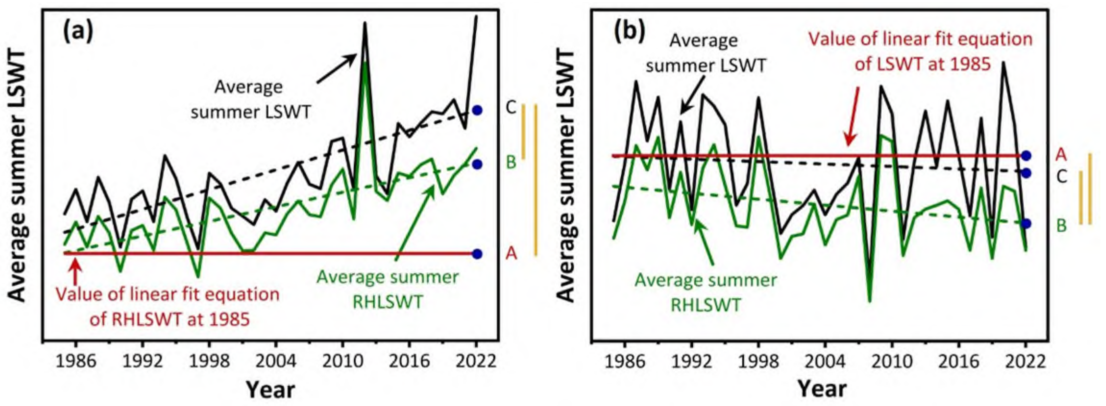

Supplementary Figure 11| Calculation of contribution. a, Positive LSWT trend. **b**, Negative LSWT trend. The black and green curves denote the average summer LSWT and RHLSWT, respectively. The dashed lines indicate the linear functions fitted from the time series of LSWT and RHLSWT, where the slopes and intercepts were calculated using the Theil-Sen method. The red solid line indicates the initial temperature, i.e., the fitted values of the linear functions of RHLSWT **(a)** and LSWT **(b)** in 1985. The blue dots indicate the corresponding values of the fitted functions in 2022. For LSWT with a positive trend, the contribution is calculated as BC/AC; for a negative trend, the contribution is calculated as BC/AB.

Supplementary Table 1| Regional average of trend in lake surface air temperature (SAT), SAT after removal of heat extremes (RHSAT), LSWT, LSWT after removal of heat extremes (RHLSWT), extreme heat of SAT, and the contribution of heat extremes.

	**Trend of SAT (°C/decade)**	**Trend of RHSAT (°C/decade)**	**Trend of LSWT (°C/decade)**	**Trend of RHLSWT (°C/decade)**	**Total heat extremes (day)**	**Trend of heat extremes (day/decade)**	**Contribution (%)**
Eastern Plain Lake Region	0.32	0.24	0.23	0.18	453.57	2.11	33.46
North-east Plain Lake Region	0.30	0.24	0.19	0.15	439.40	1.76	34.21
Inner Mongolia-Xin Jiang Lake Region	0.40	0.30	0.22	0.17	449.95	2.17	34.56
Tibetan Plateau Lake Region	0.21	0.17	0.08	0.06	472.20	1.06	40.94
Yunnan-Guizhou Plateau Lake Region	0.27	0.19	0.16	0.11	473.61	3.52	31.85
China	0.29	0.22	0.16	0.13	459.39	1.75	36.47

Supplementary Table 2| Six lakes with *in situ* observations.

Lake Name	Hylak_id	Sample size	Temporal frequency
**Lake Taihu**	148	9289	Daily
**Lake Luguhu**	15431	1014	Daily
**Lake Namco**	149	330	Daily
**Lake Hulunhu**	123	131	Monthly
**Lake Qiandaohu**	1467	1062	Daily
**Lake Erhai**	1479	12	Irregular
